# Fruit-Derived Anthocyanins: Effects on Cycling-Induced Responses and Cycling Performance

**DOI:** 10.3390/antiox11020387

**Published:** 2022-02-15

**Authors:** Cândice L. K. Copetti, Fernando Diefenthaeler, Fernanda Hansen, Francilene G. K. Vieira, Patricia F. Di Pietro

**Affiliations:** 1 Nutrition Post-Graduate Program, Department of Nutrition, Federal University of Santa Catarina, Florianopolis 88040900, Brazil; candice.lkc@posgrad.ufsc.br (C.L.K.C.); fernanda.hansen@ufsc.br (F.H.); francilene.vieira@ufsc.br (F.G.K.V.); 2 Biomechanics Laboratory, Department of Physical Education, Federal University of Santa Catarina, Florianopolis 88040000, Brazil; fernando.diefenthaeler@ufsc.br

**Keywords:** anthocyanins, supplement, exercise, performance, sports nutrition

## Abstract

Previous evidence has shown that the consumption of fruit-derived anthocyanins may have exercise benefits. This review aimed to summarize the effects of fruit-derived anthocyanins on cycling-induced responses and cycling performance. Medline, Science Direct, Cochrane Library, and SPORTDiscus online databases were searched. Nineteen articles met the inclusion criteria. The fruit-derived anthocyanins used in these studies were from cherry (*n* = 6), blackcurrant (*n* = 8), pomegranate (*n* = 2), açai (*n* = 1), and juçara fruit (*n* = 2), and were offered in juice, pulp, powder, freeze-dried powder, and extract form. The supplementation time ranged from acute consumption to 20 days, and the amount of anthocyanins administered in the studies ranged from 18 to 552 mg/day. The studies addressed effects on oxidative stress (*n* = 5), inflammation (*n* = 4), muscle damage (*n* = 3), fatigue (*n* = 2), nitric oxide biomarkers (*n* = 2), vascular function (*n* = 2), muscle oxygenation (*n* = 2), performance (*n* = 14), substrate oxidation (*n* = 6), and cardiometabolic markers (*n* = 3). The potential ergogenic effect of anthocyanin supplementation on cycling-induced responses seems to be related to lower oxidative stress, inflammation, muscle damage, and fatigue, and increased production of nitric oxide, with subsequent improvements in vascular function and muscle oxygenation leading to improved performance. In addition, the observed increase in fat oxidation can direct nutritional strategies to change the use of substrate and improve performance.

## 1. Introduction

Anthocyanins are a subclass of flavonoids [1]. The word anthocyanin comes from the Greek words (anthos, a flower, and kyanos, dark blue) and after chlorophyll, anthocyanins are the most important group of pigments of plant origin [2]. Anthocyanins are present in several vegetables and can be found in all parts of a plant, especially flowers and fruits. These pigments have a coloration that varies from red to blue, and may also appear as a mixture of both colors resulting in purple shades [3]. Anthocyanin is in the form of glycoside while anthocyanidin is known as the aglycone [4]. The anthocyanins are conjugated anthocyanidins and six of them (i.e., pelargonidin, cyanidin, delfinidin, petunidin, peonidin, and malvidin) are commonly found in berries [5], which differ in number of hydroxyls and the degree of methoxyls [6]. Glycoside formation and the greater presence of methoxyl groups generally give a more reddish color and greater stability to oxidation and heat than increased hydroxylations, which in turn provide a predominance of pink and blue [7].

Previous evidence has shown that consumption of juices/pulps/powders/extracts derived from anthocyanin-rich fruits, such as cherry, blackcurrant, pomegranate, blueberry, grape, açai, and juçara fruit may have exercise benefits due to a regulation of oxidative stress parameters [8,9,10,11,12,13,14,15], inflammation [8,9,10,15,16,17,18], muscle damage [8,16,19,20], fatigue [11,21,22], substrate oxidation [12,23,24,25], and performance [9,15,18,19,21,22,23,24,26,27,28,29,30].

Among the several types of exercise, cycling is one of the most studied modalities in the field of exercise physiology and nutrition, due to its high reproducibility of field conditions on a cycle ergometer [31]. Cycling is also among the sports activities with the largest number of practitioners in the world [32,33], with a substantial increase in the last years not only by professional athletes but also by many sportsmen and amateur athletes [34]. Cycling is a very demanding sport, where training and tests are long and lasting courses, and also with varying degrees of difficulty and intensity. In some stages of this sport, the intensity of the exercise is very high. Thus, it is considered a sport of high physical and nutritional demand, and nutrition is an essential tool in sports practice [35].

In view of the physical demands of cycling and considering the positive effects of anthocyanin supplementation observed in previous studies in the context of exercise, it is likely that the remarkable properties of anthocyanins may be useful for improving cycling performance. To the best of our knowledge, the present review is the first study that aims to summarize the effects of fruit-derived anthocyanins on cycling-induced responses and cycling performance at different intensities.

## 2. Research Strategy

**Figure 1:**
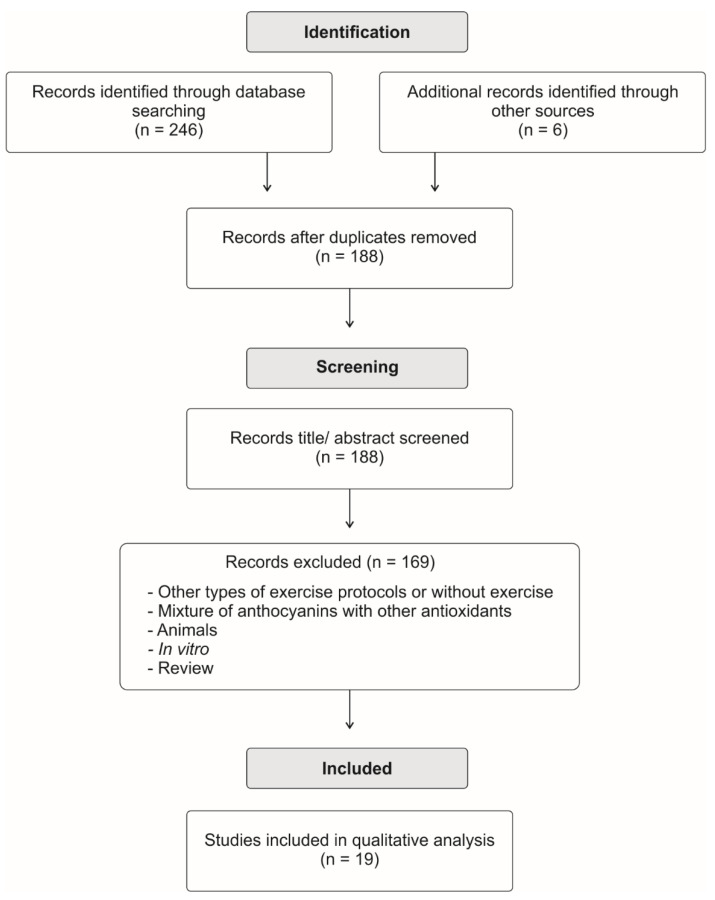
Flow diagram of the literature search process.

Bibliographic searches were performed on the Medline (via PubMed), Science Direct (via Scopus), Cochrane Library, and SPORTDiscus online databases. The combined keywords used as search descriptors were: “anthocyanin”, “exercise” and “cycling”. Boolean keys “AND” and “OR” were used. The inclusion criteria were trials that evaluated the effects of fruit-derived anthocyanins on cycling-induced responses and cycling performance without the restriction of date and written language. The titles and abstracts of all articles were read and revised and, when addressing the desired theme, the article was read in full and those that met the inclusion criteria were selected. The reference list of the eligible articles was evaluated for additional articles. A flow diagram of the literature search process is shown in Figure 1.

## 3. Fruit-Derived Anthocyanins on Cycling-Induced Responses and Cycling Performance

Nineteen articles met inclusion criteria. The fruit-derived anthocyanins used in these studies were cherry (*n* = 6), blackcurrant (*n* = 8), pomegranate (*n* = 2), açai (*n* = 1), juçara fruit (*n* = 2), and were offered in juice, pulp, powder, and extract (capsule) form. Supplementation/duration protocol ranged from acute consumption to 20 days and the amount of anthocyanins administered in the studies ranged from 18 to 552 mg/day (mean: 245.36 ± 178.26), in a daily amount of concentrated juice (60 to 250 mL), pulp (400 g), powder (6 g), freeze-dried powder (6 capsules), and extract (300 mg to 1000 mg = 1 to 3 capsules). Fifteen studies (79%) were carried out with men only, three with men and women, and one with women only. Two studies were carried out with untrained individuals, eight with recreationally trained individuals, eight with trained individuals, and one with professional athletes-classification of training status according to De Pauw et al. [36]. The articles that did not present the VO_2max_, the values were estimated using the equation proposed by Santilla et al. [37]. The sample size ranged from 8 to 26 participants (mean: 13 ± 5). Fifteen studies were conducted in the United Kingdom, three in Brazil, and one in Spain. The studies addressed effects on oxidative stress (*n* = 5), inflammation (*n* = 4), muscle damage (*n* = 3), fatigue (*n* = 2), nitric oxide biomarkers (*n* = 2), vascular function (*n* = 2), muscle oxygenation (*n* = 2), performance (*n* = 14), substrate oxidation (*n* = 6), and cardiometabolic markers (*n* = 3) (Table S1).

### 3.1. Oxidative Stress, Inflammation, Muscle Damage, and Fatigue

The effects of anthocyanins on oxidative stress, inflammation, muscle damage, and fatigue were examined in seven studies [8,11,12,15,17,18,38]. In two studies with trained cyclists equally divided into two groups: Montmorency tart cherry concentrate (MC) (2x − 30 mL + 100 mL of water − 547 mg anthocyanins (ACN) [8] and 552 mg ACN [17]) or placebo, differing only in time of supplementation: 7 consecutive days (4 days pre- and on each trial day) [8] and 8 consecutive days (4 days pre-trial, on the day of, and 3 days post-trial) [17] cyclists completed a 109 min cycling trial designed to replicate road race demands. Lipid hydroperoxides responses attenuated in one of the studies in MC group [8], while there were no differences in the other study [17]. The pro-inflammatory markers interleukin-6 (IL-6) and high-sensitivity C-reactive protein demonstrated lower values in the MC group in both studies. No group or interaction effects were found for muscle damage as measured by creatine kinase in both studies. On the other hand, recreationally trained cyclists who consumed pomegranate extract (2 capsules, amount of ACN not reported) for 15 days [38] and performed exercise tests that consisted of endurance bouts (square-wave endurance exercise test followed immediately by an incremental exercise test to exhaustion) and an eccentric exercise drill had lower creatine kinase and C-reactive protein 72 h after the trial.

Another study that evaluated the effect of juçara fruit juice consumption (250 mL − 186 mg ACN) on the inflammatory response 1 h prior to the test to high-intensity intermittent exercise in recreationally trained men observed IL-6 levels were above the baseline at 30 min and 60 min for the control (water) but were unaffected by juçara fruit juice. Interleukin-10 (IL-10) was higher in juçara fruit juice than in the control at 30 min. Tumor necrosis factor-alpha (TNF-α) was below baseline at 30 min for the control and 60 min for the juçara fruit juice. Cortisol increased above the baseline at 30 and 60 min in control whereas, for juçara fruit juice, the cortisol levels were significantly higher than the baseline at 30 min [18].

Three other studies evaluated oxidative stress biomarkers. One conducted with untrained participants who consumed MC (2x − 30 mL + 100 mL of water − 540 mg ACN) or placebo for 20 days measured total antioxidant status (TAS) pre- and post-exercise to ascertain the acute differences induced by 1 h of FATMAX exercise (intensity eliciting maximal fat oxidation rate, conveyed as a percentage of maximal oxygen uptake—VO_2max_) on a cycle ergometer at 70 rev·min^−1^ at an initial intensity of 30 W with increments of 10 W every 3 min until respiratory exchange ratio exceeded 1 for a continuous period of 30 s, and pre-, mid- and post-supplementation. Pre-exercise TAS was lower from mid- to post-supplementation with MC, suggesting that TAS does not increase linearly with additional MC intake after 10 days of returning to baseline [12]. However, after supplementation with açai pulp (400 g − 284.4 mg ACN) for 15 days, an increase in TAS and a reduction in serum lipid peroxidation (malondialdehyde) were observed in recreationally trained cyclists immediately after training (incremental test). Açai pulp supplementation was not able to reduce DNA damage, whereas after placebo intervention, an increase in the intensity of the comet tail, assessed by alkaline comet assay, was observed, indicating that there was DNA damage. These results suggest possible prevention of increased DNA damage intensity with açai pulp [15]. Another study [11] was carried out with recreationally trained men assigned to drink juçara fruit juice (250 mL − 185 mg ACN) or water (control) 1 h before a high-intensity interval training session. It was observed a decrease in oxidative stress index immediately post-exercise and an increase in reduced glutathione 1 h post-exercise. Juçara fruit juice also increased plasma total phenols and uric acid over time. In addition, lower fatigue was also observed. Fatigue was also lower with pomegranate extract in recreationally trained cyclists who performed tests that consisted of endurance bouts (square-wave endurance exercise test followed immediately by an incremental exercise test to exhaustion) and an eccentric exercise drill [38].

The studies that evaluated oxidative stress, inflammation, muscle damage, and fatigue observed improvement of oxidative stress, such as reduced oxidative stress index [11] and lipid peroxidation [8,15], and increased reduced glutathione [11], total phenols [11], uric acid [11], and TAS [12,15]; inflammation, such as reduced IL-6 [8,17], C-reactive protein [38], and TNF-α [18], and increased IL-10 [18]; muscle damage with creatine kinase reduction [38]; and fatigue, with lower fatigue [11,38]. These effects were observed in untrained [12], recreationally trained [11,18], and trained [8,15,17,38] individuals who performed high-intensity cycling intervals [8,11,17,18], incremental test [15], and square-wave endurance test followed by an incremental stress test until exhaustion and a subsequent eccentric exercise [38].

### 3.2. Nitric Oxide Biomarkers, Vascular Function, Muscle Oxygenation, and Performance

The effects of anthocyanins on nitric oxide biomarkers, vascular function, muscle oxygenation, and performance were examined in 11 studies [15,18,23,28,29,30,38,39,40,41,42,43]. Two studies evaluated nitric oxide biomarkers and vascular function. In the investigation with MC, trained cyclists ingested MC (60 mL + 100 mL of water − 73 mg ACN) or placebo 1.5 h prior to completion of 6 min moderate and severe-intensity cycling bouts. There were no significant differences in nitrate and nitrite between MC and placebo conditions, but systolic blood pressure was lower 1.5 h post MC supplementation. There were also no significant differences in time to exhaustion during the exercise tolerance test between MC and placebo conditions, but peak power over the first 20 s and total work completed during the 60 s all-out sprint was higher in MC [28]. In the study carried out with pomegranate extract, professional cyclists ingested pomegranate extract or placebo 2.5 h prior to completing a cycling time trial to exhaustion at 100% of VO_2max_ at sea level and 1657 m of altitude. Plasma nitrate was greater following pomegranate extract (1 capsule − 18 mg ACN) compared to placebo. Systolic blood pressure was not significantly affected by pomegranate extract. However, there was a trend towards an increase in systolic blood pressure with pomegranate extract versus placebo. There was a significant altitude x treatment interaction for oxygen consumption (VO_2_) and carbon dioxide production to time trial to exhaustion at 100% of VO_2max_ with pomegranate extract, although these changes were not sufficient to produce an ergogenic effect during a 100% trial to exhaustion in highly trained athletes and a longer period of supplementation may be necessary [39].

Another two studies evaluated the effects of MC on performance with freeze-dried powder in capsules. Trained competitive cyclists who consumed capsules of finely powdered freeze-dried MC (6 capsules − 257 mg ACN) or placebo for 7 days (before completion of 10 min steady-state cycling at ~65% of the VO_2 peak_ followed by a 15 km time trial) showed a time trial completion time faster and blood lactate significantly higher in MC supplementation compared to the placebo condition. Baseline tissue oxygenation index was significantly higher with MC and the difference in tissue oxygenation index between placebo and MC trials was negatively correlated with steady-state relative exercise intensity (i.e., percentage of VO_2peak_), suggesting that the increase in tissue oxygenation index after MC supplementation was more pronounced at lower exercise intensities, presumably due to the vasoactive and antioxidative effects of the phytochemicals within the MC [29]. A study carried out with recreationally trained cyclists and triathletes who consumed Cherry Active^®^, Pycnogenol^®^ (with added bioflavonoids) (1 capsule-amount of ACN not reported) or placebo 2 days before and on the day of each experimental trial, that consisted of four 5 min stages at 40%, 50%, 60%, and 70% of the maximal power output followed by a 20 km cycling time trial showed no significant differences in lactate concentrations and final 20 km time trial times [40].

Five studies carried out with New Zealand blackcurrant (NZBC) or placebo for 7 days evaluated performance-related variables. Two studies had an exercise protocol of 30 min cycling (3 × 10 min at 45%, 55%, and 65% of VO_2max_), followed by a 16.1 km time trial [23,41], but one of them had the time trial at a simulated altitude of ~2500 m (~15% O_2_) [41]. In another study, the protocol consisted of an incremental intensity cycling test followed by 10 min cycling at 65% VO_2max_ ending with the 16.1 km time trial. Between each test, participants rested for 15 min [43]. Another study was performed with 2 × 4 km time trials separated by 10 min of active recovery at the selected auto cycling intensity [30] and one with two incremental cycling protocols with the recording of physiological and cardiovascular responses [42]. NZBC (1 capsule − 105 mg ACN) reduced completion cycling time trial, coupled with a trend for higher power across the time trial and the lactate was higher with NZBC extract immediately following the time trial [23]. The total time of the two 4 km cycling trials was faster with NZBC (1 capsule − 105 mg ACN) and there was no effect of NZBC on lactate values at identical time points [30]. Plasma lactate was lower at 40%, 50%, 60%, and 70% of the maximal power output, decreases of 27%, 22%, 17%, and 13%, respectively, with NZBC and the intensity at 4 mmol∙L^−1^ onset of blood lactate accumulation was higher with NZBC without effect on heart rate and VO_2_. Despite VO_2max_, heart rate and power output were not affected by NZBC (6 g of powder − 138.6 mg ACN), VO_2max_ was achieved with 14% lower lactate [42]. No difference was observed in the time to complete the 16.1 km time trial between NZBC (2 capsules − 210 mg ACN) and placebo in normobaric hypoxia [41]. A time difference was observed between day 1 (1701 ± 163 s) and day 4 (1682 ± 162 s) for 210 mg ACN, with an increment in average speed and time to complete the 16.1 km time trial. However, there was no difference between the other days and between conditions. There was no difference in plasma lactate, VO_2_, VCO_2_, minute ventilation, heart rate, and respiratory exchange ratio between conditions and between days [43].

Two other studies evaluated the consumption of açai pulp (400 g − 284.4 mg ACN) and pomegranate extract (2 capsules, amount of ACN not reported) in recreationally trained cyclists for 15 days. In one of the studies, consumed açai pulp or placebo daily, immediately after training. It was observed that, after açai pulp supplementation, there was a reduction of 29% in lactate levels at 300 W compared to the initial test, and 28% compared to placebo. There was no increase in power output during the incremental test. However, an increase in anaerobic threshold intensity was observed after açai pulp supplementation, which compared to placebo was at the limit of significance and the authors suggest that there is an improvement in aerobic capacity [15]. In the other study, recreationally trained cyclists consumed pomegranate extract or placebo and performed tests that consisted of endurance bouts (square-wave endurance exercise test followed immediately by an incremental exercise test to exhaustion) and an eccentric exercise drill. There was a significant difference in total time to exhaustion and the time to reach the ventilatory threshold, with greater values for the pomegranate extract [38] Regarding the performance, the latest study published in this field also observed increased relative peak power output during sprint after juçara fruit juice consumption (250 mL − 186 mg ACN) compared to control in recreationally trained men [18].

The studies that evaluated nitric oxide biomarkers, vascular function, muscle oxygenation, and performance observed improvement in some variables related to nitric oxide biomarkers, such as increased nitrate [39]; vascular function, with decreasing systolic blood pressure [28]; muscle oxygenation, with increased tissue oxygenation index [29]; and performance, such as improved lactate [15,29,41,42] and total time trial [23,30,38]. These effects were observed in recreationally trained [23,41,42], trained [28,29,30], and professional [39] individuals who performed incremental tests [15], 30 min cycling (3 × 10 min at 45%, 55%, and 65% of VO_2max_), followed by a 16.1 km time trial, 6 min cycling at moderate and severe intensity [23,41], 10 min of steady state cycling at ~65% of VO_2peak_ followed by a 15 km time trial [29], 2 × 4 km time trials separated by 10 min of active recovery at the selected auto cycling intensity [30], square-wave endurance test followed by an incremental stress test until exhaustion and a subsequent eccentric exercise [38], cycling time trial to exhaustion at 100% of VO_2max_ at sea level and 1657 m of altitude [39], and two incremental cycling protocols with the recording of physiological and cardiovascular responses [42].

### 3.3. Substrate Oxidation and Cardiometabolic Markers

Seven studies evaluated substrate oxidation and cardiometabolic markers [12,23,24,25,41,43,44], six of which were carried out with NZBC and the consumption time was 7 days. Two studies had the exercise protocol of 30 min cycling (3 × 10 min at 45%, 55%, and 65% of VO_2max_), followed by a 16.1 km time trial [23,41], but one of them had the time trial at a simulated altitude of ~2500 m (~15% O_2_) [41]. In other two studies the protocol consisted of an incremental intensity cycling test followed by 10 min cycling at 65% VO_2max_ ending with the 16.1 km time trial. Between each test, participants rested for 15 min [43,44]. No change in substrate oxidation was observed [41,43], as well as in cardiometabolic markers [41,44] with the consumption of 1 or 2 capsules of NZBC (105–210 mg ACN). An increase of 27% in fat oxidation at 65% of VO_2max_ was observed, in line with a strong trend for lower carbohydrate oxidation rate with the consumption of 1 capsule of NZBC (105 mg ACN) [23]. A significant effect for time was observed for cardiac output, stroke volume, and total peripheral resistance during submaximal exercise on day 7. However, these changes were trivial and fell within the coefficient of variation of the study design [44]. In two other studies with NZBC, participants performed 120 min cycling at 65% of VO_2max_ [24,25], but in one study, participants performed four separate 120 min cycling bouts at 65% VO_2max_, after ingesting no capsule, or one of three capsules (300, 600, or 900 mg of NZBC extract − 105, 210, 315 mg ACN) [24], while in the other participants consumed a single daily dose (2 capsules − 210 mg ACN) and performed 120 min cycling at 65% of VO_2max_ [25]. NZBC increased fat oxidation over time during the cycling bout and the mean rate of carbohydrate oxidation also tended to be lower in response to NZBC. After ingesting NZBC, higher plasma concentrations of non-esterified fatty acids and glycerol were observed in the pre-exercise. Plasma non-esterified fatty acids concentrations pre-exercise were moderately associated with average fat oxidation during exercise [25]. Dose effect was also observed for average fat oxidation (0, 300, 600, and 900 mg/day values of 0.63 ± 0.21, 0.70 ± 0.17, 0.73 ± 0.19, and 0.73 ± 0.14 g/min) and carbohydrate oxidation (0, 300, 600, and 900 mg/day values of 1.78 ± 0.51, 1.65 ± 0.48, 1.57 ± 0.44, and 1.56 ± 0.50 g/min). A percentage variation was observed for 600 and 900 mg/day of the average fat oxidation was 21.5% and 24.1%, respectively, compared to no dose. NZBC increased mean fat oxidation rates, with the calculated effect sizes indicating moderate-to-large effects, compared to no dose [24].

The consumption of MC (2x − 30 mL + 100 mL of water − 540 mg ACN) or placebo was also evaluated on fat oxidation rates and cardiometabolic markers in healthy, untrained participants who performed 1 h of FATMAX exercise. No significant differences between conditions or interactions were observed for any functional and blood-based cardiometabolic markers or fat oxidation during exercise or rest. Pre-exercise high-density lipoprotein (HDL) was significantly reduced from mid- to post-supplementation with MC and the authors suggest that administering cherry interventions longer than 10 days does not maintain elevated HDL concentrations, but rather a return to baseline. However, this finding should be interpreted with caution, since no statistically and clinically significant improvements were observed for other lipid profile markers with MC [12].

Among the studies that evaluated substrate oxidation, increased fat oxidation [23,24,25] and non-esterified fatty acids and glycerol pre-exercise was observed [25]. These findings were observed in untrained [25], recreationally trained [23], and trained [24] individuals who performed 30 min cycling (3 × 10 min at 45%, 55%, and 65% of VO_2max_), followed by a 16.1 km time trial [23] and four separate 120 min cycling bouts at 65% VO_2max_ and 120 min cycling at 65% VO_2max_ [24]. It was also observed that there was a decrease in the cardiometabolic marker HDL in untrained individuals who performed 1 h of FATMAX exercise [12].

## 4. Interpretation of Research Findings

The findings of these studies indicate that individuals who consumed juice, pulp, powder, and extract derived from anthocyanin-rich fruits, for the most part, had less oxidative stress, inflammation, muscle damage, and fatigue, and increased production of nitric oxide, with subsequent improvements in vascular function and muscle oxygenation, leading to improved performance of cycling protocols. In addition, the observed increase in fat oxidation can direct nutritional strategies to change the use of substrate and improve performance. These effects were observed with different anthocyanin supplementation strategies, training status of participants, and cycling protocols, with different duration times and intensities. However, it is noteworthy that some studies have not observed changes in some variables related to these parameters. The fruit-derived anthocyanins used in these studies were cherry [8,12,17,28,29,40], blackcurrant [23,24,25,30,41,42,43,44], pomegranate [38,39], açai [15], juçara fruit [11,18] and the supplementation protocol/duration ranged from acute consumption [11,18,28,39] to 20 days [12]. The anthocyanins most prevalent in fruits of the studies included in this review were: cyanidin3-O-glucosiderutinoside, prevalent in cherry [45], delphinidin-3-rutinoside, prevalent in blackcurrant [46], cyanidin and delphinidin glucosides, prevalent in pomegranate [47,48], and cyanidin 3-glycoside and cyanidin 3-rutinoside, prevalent in açai and juçara fruits [49,50,51,52].

An aspect that is not usually discussed in studies, but that deserves attention and can be a future perspective is the evaluation of the potential mechanisms of action of specific anthocyanins and their metabolites, which are probably the cause of many responses that act in a synergistic way [53] However, the difficulty of detecting specific mechanisms to their potential effects is highlighted due to the variety of bioactive substances present in these fruits that can have a multifactorial effect. Thus, most studies address the mechanisms exerted by anthocyanins and/or polyphenols in general. In the studies included in this review, only four discuss the mechanisms of specific anthocyanins [12,23,24,25].

Among the studies that evaluated oxidative stress the main mechanisms of action reported are: the up-regulation of endogenous antioxidant enzyme defense, which have been implicated in the prevention of lipid peroxidation (via reduction of hydrogen peroxide) and the removal of lipid hydroperoxides (via 2 electron reduction to inert alcohols and water) [8], the regulation of γ-glutamylcysteine synthetase expression by polyphenols, TAS increase and maintenance of total oxidant status, elimination process by the rapid donating of an electron to a radical of hydroxyl groups attached to its phenolic rings, reducing the oxidative damage, reducing and stabilizing chemically or inactivating free radical species [7] and decreased reactive oxygen species generation [11,15].

Regarding the studies that evaluated inflammatory markers, the possible mechanisms of action discussed are: a downstream effect of reduced cell damage through oxidative stress during exercise [8], reduction in proteolytic and lipolytic cascades that are associated with inflammation via the cyclooxygenase, prostaglandin, interleukin-6 pathway [17], action potential of IL-6 myokine, requiring a smaller amount of this myokine, probably due to the associated action of polyphenols,18 and one study without conclusive results for C-reactive protein and also for the creatine kinase muscle damage marker [38]. One of the studies that evaluated fatigue is related to the antioxidant activity of anthocyanins present in the intervention, which may have neutralized the reactive oxygen species caused by exercise [11].

In the studies that found benefits in nitric oxide biomarkers, vascular function, muscle oxygenation, and performance, the likely mechanisms may involve improved endothelial function, with increased peripheral blood flow and vessel diameter by the combined action of increased nitric oxide bioavailability; alterations in production or removal of lactate through blood flow or changes in membrane lactate transport mechanisms; an increase in either the efficiency of mitochondrial O_2_ usage or in the muscular use of ATP, affording a lower VO_2_ requirement to sustain a given work rate; activation of the enzyme sirtuin 1 by allosteric interaction, which in turn activates peroxisome proliferator-activated receptor-gamma coactivator and stimulates mitochondrial biogenesis and function, consequently modulating the respiratory function; and likely due to a reduction in reactive oxygen species production and oxidative stress which may affect the sodium-potassium pump [23,24,28,29,30,39,42].

With regard to substrate oxidation, the increase in fat oxidation may result from a combination and interaction of physiological responses from many pathways acting synergistically, including the regulation of genes for proteins or key proteins regulating lipolysis involved in the increased transport of fat oxidation through the increased release of free fatty acids into the mitochondria, protein activity and expression in adipose tissue and skeletal muscle, better availability of nitric oxide and increased peripheral blood flow due to the ability of anthocyanins to inhibit nicotinamide adenine dinucleotide phosphate oxidase [23,24,25].

## 5. Methodological Limitations

A noteworthy factor is that some studies performed polyphenol/anthocyanin restrictions prior to interventions [8,11,15,17,18,28,39,40] in order to minimize inter-individual variation in anthocyanin and polyphenol consumption, while others did not [12,23,24,25,29,30,38,41,42,43,44]. This point needs to be interpreted with caution, because, while this restriction minimizes inter-individual variation in consumption, this restriction can mitigate or enhance the effects of supplementation, decreasing ecological validity [54]. Therefore, supplementing anthocyanins with the usual diet seems to be the most appropriate scenario. In addition, in studies that quantified the anthocyanin intake, polyphenols, and other antioxidants from the usual diet, the accuracy of this information may have been influenced by under- or over-reporting. Thus, it cannot be ruled out that some participants may have consumed more polyphenols and/or anthocyanins than others. In the studies that quantified the anthocyanin intake, specifically, the mean consumption ranged from ~43 [43,44] to ~67 mg/day [24,25] and the amount of anthocyanins administered in the studies included in this review ranged from 18 [39] to 552 [17] mg/day (mean: 245.36 ± 178.26), values considerably higher than the usual intake.

Given the variability in study designs and supplementation strategies (supplementation time, amount and form, i.e., juice, pulp, powder, and capsule), the type, and the time point at which biomarkers were evaluated, it is difficult to determine the length of time that anthocyanin-rich fruits exert their positive effects and whether there is a cumulative effect by which multiple doses result in increased capacity. Anthocyanin bioavailability is relatively low since after ingestion only 12% of anthocyanins appeared in the blood [55]; however, a study found that, after an acute intake of 250 mL of blueberry juice, the anthocyanin metabolites are still present in urine 5 days after additional anthocyanin intake [56]. It is also possible to conclude that only the content of supplemented anthocyanins may not be the only responsible factor for the observed effects. Rather, it is important to take into account that the participants’ initial physiological status can also influence supplementation effectiveness, since the baseline antioxidant status is an important determinant of the ergogenic effectiveness of an antioxidant intervention, as individuals with low antioxidant status can respond better to an intervention rich in antioxidants when compared to individuals with moderate and high antioxidant status [54]. In order to assess the bioavailability of anthocyanins, future studies should be conducted under conditions in which individuals would keep their typical diet before consuming anthocyanin-rich foods and exercising, and that include the quantification of blood anthocyanins and/or polyphenols and their metabolites, such as phenolic acids, which may guide adjustments to the ideal dose to be administered, since the metabolites of anthocyanins and other polyphenols can act synergistically [57]. In addition, the quantification of blood anthocyanins and/or polyphenols and their metabolites is a way to control adherence to the intake protocol, enhancing the cause–effect relationship of the intervention protocol. We also recommend studies to compare the administration of different doses of anthocyanins in the same cycling-induced responses, in order to provide more robust evidence regarding the ideal dosage to be administered.

Finally, most studies used participants predominantly of the male sex (15/19) and the results of these studies cannot be extrapolated to females. It is also possible that some studies do not have sufficient power to allow firm conclusions about the effects of anthocyanins due to the sample size, which ranged from 8 to 26 participants (mean: 13 ± 4.15), plus the fact that several findings may also be related to differences in the intensity and duration of the exercise protocols, as well as the training status of the participants, which ranged from untrained to professional.

## 6. Conclusions

The potential ergogenic effect of supplementing juice, pulp, powder, or extract derived from anthocyanin-rich fruits on cycling-induced responses seems to be related to lower oxidative stress, inflammation, muscle damage, fatigue, and increased production of nitric oxide, with subsequent improvements in vascular function and muscle oxygenation leading to improved performance of cycling-induced responses. In addition, the observed increase in fat oxidation can direct nutritional strategies to change the use of substrate and improve performance.

## Supplementary Materials

The following supporting information can be downloaded at: https://www.mdpi.com/article/10.3390/antiox11020387/s1. Table S1: Effects of fruit-derived anthocyanins on cycling-induced responses and cycling performance.
