# Supplementary material for: Fruit-Derived Anthocyanins: Effects on Cycling-Induced Responses and Cycling Performance
Source: Antioxidants (Basel). 2022 Feb 15;11(2):387. doi: 10.3390/antiox11020387 (PMC8869496; doi:10.3390/antiox11020387)
Supplement: Supplementary file 1 [file antioxidants-11-00387-s001.zip › antioxidants-1565015-supplementary.pdf]

antioxidants

MDPI

Table S1. Effects of fruit-derived anthocyanins on cycling-induced responses and cycling performance. Captions: ACN, anthocyanins; ALT, altitude; ATi, anaerobic threshold intensity; CK, creatine kinase; CM, cardiometabolic markers; CO, cardiac output; CRP, C-reactive protein; DNA, deoxyribonucleic acid; DBP, diastolic blood pressure; F, females; FATMAX, intensity eliciting maximal fat oxidation rate, conveyed as percentage of maximal oxygen uptake (%VO_{2max}); GSH, reduced glutathione; Hct, hematocrit; HDL, high density lipoprotein; HIIE, high-intensity intermittent exercise; HIIT, high-intensity interval training; HR, heart rate; hsCRP, high-sensitivity C-reactive protein; I, inflammation biomarkers; IL-1-β, interleukin-1-beta; IL-6, interleukin-6; IL-8, interleukin-8; [La−]b, blood lactate concentration; LDL, low density lipoprotein; LOOH, lipid hydroperoxides; M, males; MAP, mean arterial pressure; MD, multiple daily; MDA, malondialdehyde; MO, muscle oxygenation; MVIC, maximum voluntary isometric contraction; NEFA, non-esterified fatty acids; NO, nitric oxide biomarkers; NO_2^-, nitrite; NO_3^-, nitrate; OS, oxidative stress biomarkers; OSI, oxidative stress index; O_2, oxygen; P, performance; RER, respiratory exchange ratio; RPE, ratings of perceived exertion; rPPOspint, relative peak power output sprint; SBP, systolic blood pressure; SD, single daily; SEA, sea level; SV, stroke volume; SO, substrate oxidation; TAS, total antioxidant status; TEAC, trolox equivalent antioxidant capacity; TNF-α, tumor necrosis factor-alpha; TOI, tissue oxygenation index; TPR, total peripheral resistance; TTE, trial to exhaustion; VCO_2, carbon dioxide produced; VE, minute ventilation; VF, vascular function; VO_2, oxygen uptake; VO_{2max}, maximal oxygen consumption; VT2, ventilatory threshold 2; ↑ increase; ↓ decrease; → maintenance/no significant differences when compared to the control group.

References	Sample	Intervention					
Authors/Year/Location/Ref nº	Population: nº and Sex, * Training Status	Supplementation Protocol/Duration	Fruit and Processing Method	Intervention Strategy/ Total Daily Anthocyanin Amount	Cycling Protocol/Duration	Blood Collection Time Points	Key Findings
Bell et al. (2014) [8]—United Kingdom	16 M, trained VO_{2max} = 61.6 ± 10.4 mL. kg. min	7 days	Cherry, juice concentrate	MD: 2x − 30 mL + 100 mL of water 547 mg ACN	High-intensity cycling intervals, 109 min	Baseline (prior to 4-day loading phase), pre- and post-trial 1, pre- and post-trial 2, and pre- and post-trial 3	**OS:** ↓ LOOH<br>**I:** ↓ IL-6 and hsCRP → IL-8, IL-1-β, and TNF-α<br>**Muscle Damage:** → CK
Bell et al. (2015) [17]—United Kingdom	16 M, trained VO_{2max} = 61.6 ± 10.4 mL. kg. min	8 days	Cherry, juice concentrate	MD: 2x − 30 mL + 100 mL of water 552 mg ACN	High-intensity cycling intervals, 109 min	Baseline (prior to 4 the day loading phase), immediately pre-trial, immediately post-trial, and 1, 3, 5, 24, 48, and 72 h post-trial	**OS:** → LOOH<br>**I:** ↓ IL-6 and hsCRP, → IL-8 and TNF-α<br>**Muscle Damage:** → CK<br>**P:** MVIC decline attenuation and ↓ VO_2
Cliford; Mitchell; Scott (2013) [40]—United Kingdom	9 M, recreationally trained VO_{2max} = 52.4 ± 8.7 mL. kg. min	3 days	Cherry, powdered freeze-dried	SD: 1 capsule Not reported	Four 5 min stages with maximum power output of 40%, 50%, 60%, and 70% (W max), followed by a 20 km time trial, ~53 min	Before the end of each stage	**P:** → Lactate and time trial
Cook et al. (2015) [23]—United Kingdom	14 M, recreationally trained VO_{2max} = 53.0 ± 6.0 mL.kg.min	7 days	Blackcurrant, extract	SD: 1 capsule 105 mg ACN	30 min of cycling (3 × 10 min at 45, 55 and 65% of VO_{2max}), followed by a 16.1 km time trial, ~ 58 min	Between each exercise stage of incremental cycling test, 4 min after the end of the of incremental cycling test, 5 min into each stage (i.e., at 5, 15, 25 min of submaximal cycling intensities), and every minute for the first 5 min, and then taken every 5 min for	**P:** ↑ Lactate<br>↓ Total time to complete the 16.1 km time trial<br>**SO:** ↑ Fat oxidation → Carbohydrate oxidation

						a total of 15 min after the 16.1 km cycling time trial	
Cook et al. (2017) [24]—United Kingdom	15 M, trained VO_{2max} = 56.7 ± 7.9 mL.kg.min	7 days	Blackcurrant, extract	SD: 1–3 capsules 105–315 mg ACN	Four separate 120 min cycling bouts at 65% VO_{2max}, 120 min	At the beginning of the rest stage of the incremental cycling test, 4 min after the end of the incremental cycling test and every 15 min (i.e., at 15, 30, 45, 60, 75, 90, 105, and 120 min) of the 120 cycling protocol	**P:** → VO_2, VCO_2, and lactate **SO:** ↑ Fat oxidation → Carbohydrate oxidation
Copetti et al. (2020) [11]—Brazil	15 M, recreationally trained ** VO_{2max} = 49.1 ± 7.2 mL. kg. min	1 day—Prior to the test	Juçara fruit, juice concentrate	SD: 250 mL 185 mg ACN	HIIT: 7 series of high-intensity cycling lasting 60 s and a workload corresponding to 100% of the peak power obtained in the maximum incremental test with a 75 s active recovery interval (30 W), 17 min	1 h prior to exercise, immediately post-exercise, and 1 h post-exercise	**OS:** ↑ GSH ↓ OSI ↑ Total phenols and uric acid (over time) **Fatigue:** ↓ Fatigue index
Crum et al. (2017) [39]—United Kingdom	7 M, 1 F, professional VO_{2max} = 74.4 ± 6.2 mL. kg. min	1 day—Prior to the test	Pomegranate, extract	SD: 1 capsule 18 mg ACN	Cycling TTE at 100% VO_{2max} at sea level (SEA) and at 1657 m of altitude (ALT) ~10 min	Before the test	**NO:** ↑ NO_3^- → NO_2^- **VF:** → SBP and DPB **P:** ↑ Hct and VO_2 at 1657 m of ALT ↓ Hct at SEA → TTE 100%, VCO_2 and $[La^-]b$
Desai et al. (2018) [12]—United Kingdom	7 M, 4 F, untrained VO_{2max} = 35.9 ± 4.8 mL. kg. min	20 days	Cherry, juice concentrate	MD: 2x − 30 mL + 100 mL of water 540 mg ACN	1 h of FATMAX exercise that consist in cycling on cycle ergometer at 70 rev·min^{-1} at an initial intensity of 30 W with increments of 10 W every 3 min. The test was terminated once RER exceeded 1 for a continuous period of 30 s	Before and after exercise	**OS:** ↓ Pre-exercise TAS **CM:** ↓ HDL, → serum glucose, triglycerides, LDL, and HR **SO:** → mean fat and carbohydrate oxidation rates
Keane et al. (2018) [28]—United Kingdom	10 M, trained VO_{2max} = 59.0 ± 7.0 mL. kg. min	1 day—Prior to the test	Cherry, juice concentrate	SD: 60 mL + 100 mL of water 73 mg ACN	6 min of cycles of moderate and severe intensity. The severe intensity test was continued until exhaustion on one occasion and immediately	Baseline, over the 20 s preceding the step transition in work rate, the 20 s preceding the completion of 360 s of moderate- and severe-intensity cycling exercise, immediately following	**P:** ↑ Peak power in the first 20 s and the total work completed during the total 60 s sprint ↑ Test peak power by 9.5% and the total work completed during the 60 s sprint by 10% between → TTE, VO_2 and lactate

					followed by a total sprint of 60 s	the 60 s all-out sprint and immediately after exhaustion during the severe-intensity constant-work-rate trial	**MO:** → TOI<br>**NO:** → NO_2^- and NO_3^-<br>**VF:** ↓ SBP
Mendes et al. (2021) [18]—Brazil	15 M, recreationally trained ** VO_{2max} = 49.1 ± 7.2 mL. kg. min	1 day—Prior to the test	Juçara fruit, juice concentrate	SD: 250 mL 186 mg ACN	HIIE: 7 series of high-intensity cycling lasting 60 s and a workload corresponding to 100% of the peak power obtained in the maximum incremental test with a 75 s active recovery interval (30 W), 17 min	Before and at 0, 30, and 60 min post-HIIE	**I:** ↑ IL-10 and cortisol<br>↓ TNF-α<br>→ IL-1-β, IL-6, and IL-8<br>**P:** ↑ $rPPO_{spint}$
Montanari et al. (2020) [43]—United Kingdom	13 M, trained VO_{2max} = 55.3 ± 6.7 mL. kg. min	7 days	Blackcurrant, extract	SD: 1–2 capsules 105–210 mg ACN	Incremental intensity cycling test followed by 10 min cycling at 65% VO_{2max} ending with the 16.1 km time trial ~28 min	During the last minute of every stage in incremental cycling test and after completion of 16.1 km	**P:** → Lactate, VO_2, VCO_2, V_E, and RER<br>A time difference was observed between day 1 (1701 ± 163 s) and day 4 (1682 ± 162 s) for 210 mg ACN, with an increment in average speed and time to complete the 16.1 km time trial. However, there was no difference between the other days and between conditions<br>**SO:** → Carbohydrate and fat oxidation rates<br>**CM:** → HR
Montanari et al. (2021) [44]—United Kingdom	13 M, trained VO_{2max} = 55.3 ± 6.7 mL. kg. min	7 days	Blackcurrant, extract	SD: 1–2 capsules 105–210 mg ACN	Incremental intensity cycling test followed by 10 min cycling at 65% VO_{2max} ending with the 16.1 km time trial ~28 min	At the end of each stage	**CM:** → SBP, DBP, MAP, HR, SV, CO, and TPR at rest<br>A significant effect for time was observed for CO, SV, and TPR during submaximal exercise on day 7. However, these changes were trivial and fell within the coefficient of variation of the study design
Morgan; Barton; Bowtell (2019) [29]—United Kingdom	8 M, trained VO_{2max} = 62.3 ± 10.1 mL. kg. min	7 days	Cherry, powdered freeze-dried	MD: 6 capsules 257 mg ACN	10 min of steady state cycling at ~65% VO_{2peak} followed by a 15 km time trial, ~36 min	Baseline, post steady-state exercise, and post time trial	**MO:** ↑ TOI<br>**P:** ↓ Total time to complete the 15 km time trial<br>↑ Lactate<br>→ VO_2 and RER
Murphy, Cook; Willems (2017)	10 M, trained	7 days	Blackcurrant, extract	SD: 1 capsule 105 mg ACN	2 × 4 km time trials separated by 10 min of active recovery at the	Immediately after each trial and 8 min into recovery between the trials	**P:** ↓ The total time of the two 4 km cycling trials by 0.82%<br>→ Lactate

[30]—United Kingdom	VO_{2max} = 55.0 ± 7.0 mL. kg. min				selected auto cycling intensity, ~6 min		
Strauss et al. (2018) [25]—United Kingdom	16 F, untrained VO_{2max} = 43.7 ± 1.1 mL. kg. min	7 days	Blackcurrant, extract	SD: 2 capsules 210 mg ACN	120 min cycling at 65% VO_{2max}	Baseline and at 15 min intervals throughout the exercise	**SO:** ↑ Fat oxidation over time during the cycling bout ↑ NEFA and glycerol pre-exercise the cycling bout Tendency to ↓ carbohydrate oxidation
Terrazas et al. (2019) [15]—Brazil	10 M, recreationally trained ** VO_{2max} = 45.5 ± 3.5 mL. kg. min	15 days	Açaí, pulp	SD: 400 g 284.4 mg ACN	Incremental test started at 150 W, with increments of 25 W every 2 min until voluntary exhaustion, protocol duration not reported	At the beginning and end of each trial, before performing the incremental test	**OS:** ↓ MDA ↑ TEAC → DNA damage **P:** ↓ Lactate ↑ATi
Torregrosa-García et al. (2019) [38]—Spain	26 M, recreationally trained VO_{2max} = 54.4 ± 9.0 mL. kg. min	15 days	Pomegranate, extract	SD: 2 capsules Not reported	Square-wave endurance test followed by an incremental stress test until exhaustion and a subsequent eccentric exercise ~105 min	At the end of the test (lactate) and 10 min before the first isokinetic test and subsequently upon each strength assessment completion (2, 24, 48, and 72 h later) for muscular damage and inflammation biomarkers	**I:** ↓ CRP **Muscle damage:** ↓ CK **Fatigue:** ↓ % work fatigue **P:** ↑ TTE and the time to reach VT2 → VO_{2max}, VO_2 at VT2, lactate RPE, and peak torque
Willems et al. (2015) [42]—United Kingdom	8 M, 5 F, recreationally trained VO_{2max} = 49.1 ± 6.2 mL. kg. min	7 days	Blackcurrant, powder	SD: 6 g 138.6 mg ACN	2 incremental cycling protocols with the recording of physiological and cardiovascular responses, protocol duration not reported	Immediately after each stage and 3 min after voluntary exhaustion during the maximum oxygen uptake test	**P:** ↓ Lactate → Power and maximum values of oxygen uptake. However, maximum oxygen uptake with blackcurrant powder was obtained with lactate values 14% lower
Willems et al. (2019) [41]—United Kingdom	11 M, recreationally trained VO_{2max} = 47.0 ± 7.0 mL. kg. min	7 days	Blackcurrant, extract	SD: 2 capsules 210 mg ACN	Steady state cycling test (3 × 10 min at 45%, 55%, and 65% VO_{2max}) followed by a 16.1 km time trial at a simulated altitude of ~2500 m (~15% O_2), ~58 min	In the last 30 s of each stage of incremental cycling test for oxygen–cycling power relationship and 3 min after the end of the incremental cycling test for maximum oxygen uptake	**P:** → 16.1 km cycling time trial, lactate, and RER **SO:** → substrate oxidation during steady state cycling at 45%, 55%, and 65% VO_{2max} **CM:** → Blood glucose and HR

* Classification of training status according to De Pauw et al. (2013) [36]. ** Estimated VO_{2max} according to Santilla et al. (2013) [37].
